# Intracranial electrophysiological recordings from the human brain during memory tasks with pupillometry

**DOI:** 10.1038/s41597-021-01099-z

**Published:** 2022-01-13

**Authors:** Jan Cimbalnik, Jaromir Dolezal, Çağdaş Topçu, Michal Lech, Victoria S. Marks, Boney Joseph, Martin Dobias, Jamie Van Gompel, Gregory Worrell, Michal Kucewicz

**Affiliations:** 1 Brain and Mind Electrophysiology laboratory, Multimedia Systems Department, Faculty of Electronics, Telecommunications and Informatics, Gdansk University of Technology, Gdansk, Poland; 2 Department of Biomedical Engineering, International Clinical Research Center, St. Anne’s University Hospital Brno, Brno, Czech Republic; 3 Czech Institute of Informatics, Robotics and Cybernetics, Czech Technical University in Prague, Prague, Czech Republic; 4 Department of Neurology, Mayo Clinic, Rochester, MN USA; 5 Department of Physiology & Biomedical Engineering, Mayo Clinic, Rochester, MN USA; 6 Faculty of Electrical Engineering, Czech Technical University in Prague, Prague, Czech Republic; 7 Department of Neurosurgery, Mayo Clinic, Rochester, MN USA

**Keywords:** Sensory processing, Consolidation, Hippocampus, Cortex

## Abstract

Data comprise intracranial EEG (iEEG) brain activity represented by stereo EEG (sEEG) signals, recorded from over 100 electrode channels implanted in any one patient across various brain regions. The iEEG signals were recorded in epilepsy patients (N = 10) undergoing invasive monitoring and localization of seizures when they were performing a battery of four memory tasks lasting approx. 1 hour in total. Gaze tracking on the task computer screen with estimating the pupil size was also recorded together with behavioral performance. Each dataset comes from one patient with anatomical localization of each electrode contact. Metadata contains labels for the recording channels with behavioral events marked from all tasks, including timing of correct and incorrect vocalization of the remembered stimuli. The iEEG and the pupillometric signals are saved in BIDS data structure to facilitate efficient data sharing and analysis.

## Abstract (summary)

**Table:** 

Measurement(s)	intracranial electroencephalogram measurement • gaze • pupil size trait
Technology Type(s)	intracranial electroencephalography (iEEG) • gaze tracking • Pupillometry
Factor Type(s)	patient • task
Sample Characteristic - Organism	Homo sapiens

Machine-accessible metadata file describing the reported data: 10.6084/m9.figshare.16847566

## Background & Summary

Intracranial EEG (iEEG) monitoring is a standard diagnostic procedure used to localize and treat sources of epileptogenic activities in patients suffering from pharmacoresistant focal epilepsy^1^. The iEEG signals are acquired directly from brain structures using various types of implanted electrode contacts, ranging from standard clinical macro-contacts to special micro-contacts. Apart from their clinical value, the acquired signals present a unique opportunity to investigate physiological processes in the human brain, including those related to memory and cognition^2–4^, at a large scale of neural activities^5^. Most of the studies employing these signals in cognitive tasks are limited by clinical and research recording systems, multiple formats for acquiring and storing data files, and separate data structures for electrophysiological, behavioral, pupillometric, and brain imaging data.

The iEEG recording systems used in the clinical setting are typically limited to low sampling rates and inefficient compression formats^6^. Systems used for research recordings offer excellent sampling rates of up to 40 kHz and efficient compression formats to store continuously sampled signals from hundreds of channels over multiple days of the seizure localization procedure^6,7^. This allows large-scale recordings of electrophysiological processes sampled both at the macro- and at the micro-level of brain organization^2,5,8^. However, these large-scale recordings, which require high temporal (sampling rate) and spatial (size and number of channels) resolution, grow rapidly in size. The large-scale iEEG recordings present a challenge for efficient storing, organization, and processing^6,7^. In addition, these rare iEEG datasets need to be synchronized with data from behavioral tasks and with brain imaging data for electrode localization. All these various sources of data could ideally be stored in one environment with a structure universal for various types of brain signals and tasks. Recently, the widely-recognized Brain Imaging Data Structure (BIDS)^9^ was proposed for a universal organization of iEEG data^10^ that would accommodate the large scale of electrophysiological signals sampled in any behavioral task.

Well organized datasets are critical for rapid sharing and exploration of the iEEG data and open the way for large datasets with recordings collected from national and international clinical centers^10^. The largest database of iEEG recordings during memory tasks was collected in a BRAIN initiative project called Restoring Active Memory^11^ with recordings collected from over 300 patients at multiple clinical centers. Although the project yielded a whole series of seminal papers about human memory enhancement^12–14^ and the neural mechanisms of memory processing^15–18^, the dataset revealed multiple challenges for use of this and similar datasets by researchers outside of such projects. One challenge is related to combining anatomical data for electrode localization^19^. Others are related to new structures and formats used in such datasets that require various custom-made toolboxes for processing the data. Marking events and metadata in the data signals presents yet another challenge that is addressed by unified database structures^10^. Adding complementing behavioral data, e.g. from pupillometry 20, introduce another challenge for organizing the datasets^20^ in a unified structure.

Therefore, there is a growing demand for new open-access and universal datasets with the human iEEG task recordings. There are several recent reports using large-scale iEEG recordings of ECoG, local field potential, and single unit spiking activities during memory tasks^21–23^ that could be cross-validated or reproduced in one combined large database from these various tasks. In addition, the same findings that were observed for a given frequency range (70–150 Hz) could be reproduced e.g. in the higher ripple and fast ripple frequency bands or in the single and multi-unit activity ranges (>1000 Hz), where other memory-associated activities were reported^24–27^. Currently, these datasets are limited to only a selected range of these activities instead of a broad scale of wide-bandwidth signals.

The purpose of sharing this dataset is to provide researchers with high quality and accessible recordings of iEEG signals during tasks stored in a universal structure together with pupillometry signals. This will help to advance the understanding of the multiple brain activities and cognitive processes reflected in the eye movements that are engaged during memory performance. Here, we aim to combine measures from large-scale electrophysiology and cognitive psychology in one universal structure for efficient analysis and sharing to accelerate novel discoveries and deepen the knowledge about human memory and the associated brain functions.

## Methods

### Ethics declaration

Data were collected from patients at Mayo Clinic in accordance with the research protocol approved by the local IRB (15-006530). Informed consent was obtained from each participant and all procedures were performed in accordance with the relevant guidelines and regulations approved by the Mayo Clinic IRB.

### Data collection

We used intracranial recordings made during free recall memory tasks in patients undergoing iEEG monitoring as part of clinical treatment for drug-resistant epilepsy. Electrophysiological signals were collected from electrodes implanted into the brain parenchyma with 32 kHz sampling frequency. The signals were collected from standard clinical penetrating depth electrodes (AdTech Inc., PMT Inc.) implanted into the brain parenchyma. The depth electrode contacts were separated by 5–10 mm spacing. The patients were implanted with the depth electrodes using the stereo EEG (sEEG) procedure, which involved stereotactic planning of implantation trajectories in 3D anatomical space of the brain. Note that the ECoG data are not published within the here presented version of this dataset but will be added in the future versions. For each patient, the number and placement of the electrodes were determined by a clinical team with the goal of localizing epileptogenic brain regions as part of the patient’s evaluation for epilepsy surgery. The postimplantation anatomical location of electrode contacts was determined by expert reviewers. The anatomical terminology followed the ‘Atlas of the Human Brain’^28^

**Fig. 1:**
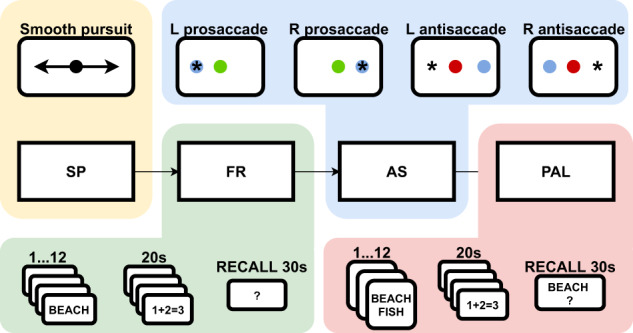
Overview of the battery of tasks as performed by the patients. SP – smooth pursuit, FR – free recall, AS – anti-pro saccade, PAL - paired-associate learning.

Following electrode implantation, each patient participated in a battery of four cognitive tasks, probing verbal memory and eye movements with tracking of pupil size and gaze position on the task display screen. All tasks were performed by the implanted patients resting comfortably in a hospital bed within one experimental task session a day. Task events were annotated automatically in the iEEG recordings with TTL pulses generated in the task computer and transmitted to a parallel input port for the iEEG signal amplifier (Neuralynx Inc., Bozeman MT). Behavioral responses (vocalizations of remembered word stimuli and eye movements on the screen) were processed after the experiment using microphone recordings and the pupillometric signals acquired on the task computer (i4tracking system, Medicton Group Ltd., Prague, Czech Republic). Task calibration, progression through the trials, and switching between the tasks were operated by the experimenter on the task computer without any need for patients to press the keyboard or make any motor movement except for the eye movements and the vocalizations. Patients were instructed to lay still in the bed, refrain from moving, and focus their gaze on the center of the screen. Only the task monitor was presented in front of the patients. Position of the monitor for optimal pupil detection was determined by visually checking real-time pupil detection before starting the task battery. In addition, an automatic eye tracking calibration check was conducted between the tasks to ensure high quality of the pupillometry data. The general flow of one task session is depicted in Fig. 1.

The battery started with a Smooth Pursuit (SP) task where patients follow a horizontally moving dot in the middle of the screen with constant slow acceleration (Fig. 1). SP controls not only for any oculomotor and/or sensory deficits but also for sensorimotor (non-verbal) working memory with known neurophysiological substrates impaired in specific brain disorders^29^.

The SP task was followed by the main verbal memory task for Free Recall (FR) of word lists^30^, in which patients were asked to remember 12 words presented one at a time on a computer screen, and then to recall them in any order after a short distractor task with simple algebraic equations, as we described before in Kucewicz *et al*. (2017)^17^ (Fig. 1). In each complete FR session there were 15 such word lists (180 different word epochs in total). Patients were free to stop the task at any point. Interictal epileptiform activities^31–33^ and seizures, including micro-seizures^8^, were previously reported in these recordings and could affect performance in the tasks. In contrast to the previous studies with the FR task, we repeated the same word lists on subsequent FR sessions on the following days of the hospital stay at regular intervals (24 and 72 hours after the first FR session). Thus, the same word lists were used for a patient and presented in the same order to enable study of reinstatement of specific electrophysiological patterns during the task and their reactivation during delayed recall in the task^34–37^.

After the FR task, another eye movement task was administered to reduce interference of the word stimuli before the final task using the same pool of common English nouns. This task is a classic paradigm for saccadic and anti-saccadic (AS) eye movements^38^ directed toward a target colored dot shifting position either to the right or to the left side of the screen center (Fig. 1). AS was used to probe non-verbal sensorimotor working memory for holding information about the color of the presented dot, which determines either a pro-saccade (green), i.e. follow the coming shift to either side, or an anti-saccade (red), i.e. do not follow the shift and saccade to the opposite side. There were 120 trials of each type that were pseudo-randomly distributed between two blocks of 60 trials with equal number of the pro- and anti-saccade trials. Like the SP task, AS was described with a well-defined network of the underlying neural substrates and mechanisms, which are impaired in a range of brain disorders^39^.

The battery was concluded with another version of the verbal memory task that used 6 pairs of words displayed one after another for subsequent cued recall (in contrast to the list of 12 words presented for non-cued free recall in FR), following the same distractor delay as in FR, to probe paired-associate learning (PAL)^30^. In PAL, the 15 lists of word pairs are initially presented in the same order across the sessions, but the order of the paired associate words (one word from each pair) during the cued recall phase is randomized. In this recall stage, one of the words from each pair (randomly selected) is presented as the cue for recall (Fig. 1). This design was employed to control for any effects of the predicted sequence of words, as in the previous studies^22,37^. The same pool of common English nouns was used for both FR and PAL tasks (randomly assigned for each list without replacement) to enable investigation of the iEEG activities in response to the same words in the context of two different tasks. Overall, within any session each of the 180 words was presented at least 2 times. There was at least one arithmetic equation presented per each list for control comparison of the word-induced iEEG responses.

In summary, each task session comprised a battery of the four tasks administered in the following order: SP, FR, AS, PAL. The aim was to record all tasks during one task session but this was not always possible. The recordings were collected from a minimum of one daily task session with at least one task completed (Table 1).

**Table 1:** Overview of the total number of tasks and electrode recordings in individual patients.

	Subject	AP runs	FR runs	PAL runs	SP runs	N contacts	Vision aids	Interpupillart distance (mm)	Psychiatry
1	sub-003	1	2	0	1	128	No	75	depression, ADHD
2	sub-004	2	2	2	2	128	No	65	No
3	sub-005	4	4	4	4	128	No	65	No
4	sub-006	0	1	1	0	128	No	64	No
5	sub-007	1	1	1	1	128	No	68	No
6	sub-008	0	0	0	1	128	No	65	No
7	sub-009	0	2	2	2	70	No	61	No
8	sub-010	0	1	0	1	118	Glasses	60	No
9	sub-011	2	2	2	2	128	Glasses	65	depression
10	sub-012	3	4	4	3	128	Contact lenses	58	No
	Total N	13	19	16	17	1212			

Different runs of the same task peformed by the same patient are separated by 24–72 hours.

### Tracking of eye movements and pupil dilation

Recording of gaze position and pupil size was performed using a custom-made application developed from the i4tracking system (Medicton group Inc., Prague, Czech Republic) designed for clinical use^40^. The recording was performed on a laptop computer connected to a 24-inch monitor screen with resolution of 1680 × 1050 px, where the gaze position was tracked by high-resolution (2048 × 1088 px) and high-speed (up to 150 Hz sampling rate) external camera device placed right under the screen in front of the patient. Stimuli were displayed on the screen using font size of 100 pt and were viewed from a distance of approx. 60 cm. Pupil position and size were detected by the camera device, corresponding to approx. 0.1 mm per pixel in the eye image. The camera device was positioned to capture the face area from forehead to the mouth. Two sources of infrared light were emitted from the camera to capture the reflected light for pupil detection. Other sources of infrared light in the room were eliminated. Raw images from the camera were sampled at the rate of 150 Hz and were saved for extracting pupil size and position using a detection algorithm, which worked by fitting a general ellipse equation over the estimated pupil image. The pupil size in pixels was also converted to millimeters using measured interpupillary distance (IPD) and the IPD in the camera images. The reported pupil area was computed using the corresponding vertical and horizontal diameters in the ellipse area equation. Gaze position was determined by projecting the movement of the estimated center of the pupil onto the monitor screen area with the use of corneal reflection. Gazes outside of the screen area as well as the eye-blinks were treated as missing-samples. Vocal responses of the patients during the recall phase of the task were recorded using an external USB microphone and manually annotated after the experiments to mark the time and identity of the uttered words in custom audio editing software.

Before presentation of the task word lists, the eye tracker was calibrated for each recruited patient. In the calibration procedure patients were asked to focus their gaze on nine points presented consecutively at specific positions across the diagonals and centers of the side edges of the display screen. Calibration was repeated before starting any new task in the session to ensure accurate estimate of the pupil size. Moreover, patients were instructed not to move their heads and focus gaze on the screen throughout all phases of the task trials. Despite these efforts, the quality of the eye-tracking was lower toward the end of each task due to slight shifts in the focus with changes in the head position. All stimuli were presented on the screen in a light gray color on light gray background to minimize pupil responses to changing lighting and contrast. The testing was conducted in a room with low-light conditions that remained constant throughout the testing procedure.

### Format conversion

**Fig. 2:**
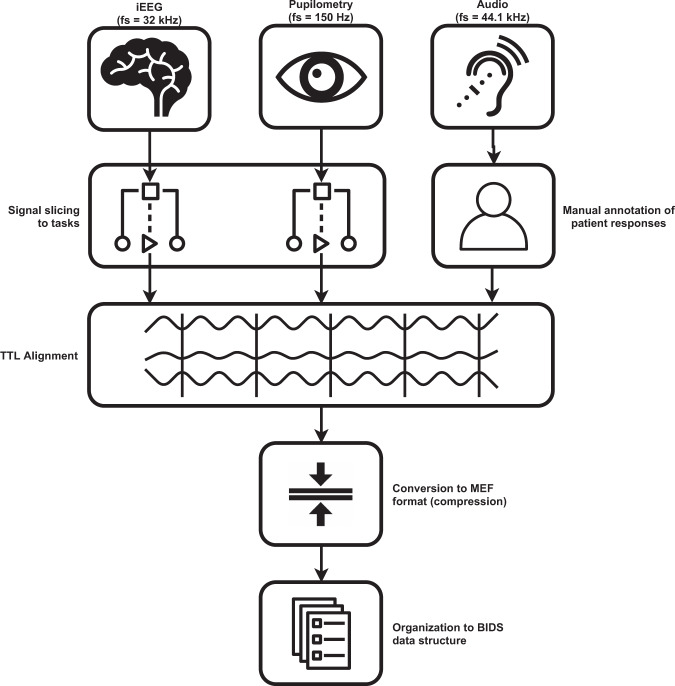
Flowchart of data conversion to the final structure.

**Fig. 3:**
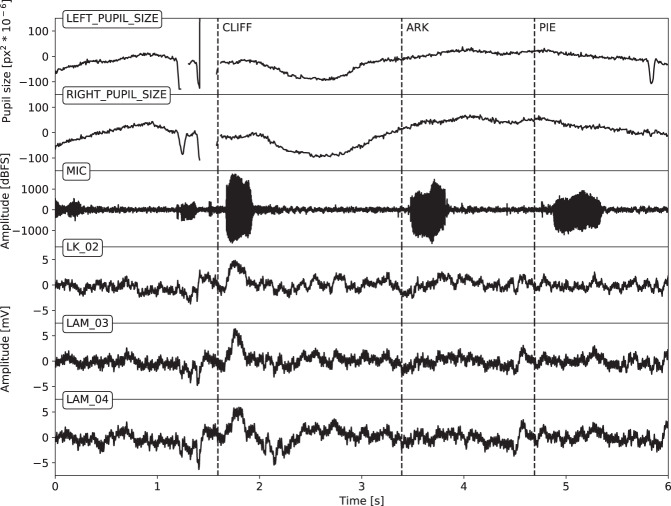
Example data showing time synchronization of signals. The vocal (fs = 44.1 kHz), pupillometric (150 Hz) and iEEG (fs = 32 kHz) signals are presented. The dashed lines represent manual annotations of the start of vocalizing recalled words in FR task.

The time series data (Nuralynx iEEG, i4tracking pupillometry and audio) were processed by a software pipeline (Fig. 2), aligned in time based on TTL pulses that indicated specific annotated events in the tasks (Fig. 3), stored in Multiscale Electrophysiology Format (MEF) version 3^6^, and organized in BIDS data structure for further viewing and automated processing. All data were de-identified during the conversion process by omitting any information that could lead to identification of the patient, such as name and date of birth.

## Data Records

Currently the data comprises recordings from 10 patients with 65 tasks performed. Summary of the tasks performed with recordings from a specified number of implanted contacts is presented in Table 1.

The raw time series data (iEEG, audio, pupillometry) were stored in the MEF3 format^6^ due to its capability for handling data compression, variable sampling frequency across signals, and any signal discontinuities (Fig. 3). The MEF C library is publicly available at https://github.com/msel-source/meflib. The high level API readers exist for python (https://github.com/msel-source/pymef) and for Matlab (https://github.com/MaxvandenBoom/matmef). Overview of the processing structure is summarized in Fig. 2.

The data and metadata are organized in BIDS data structure for iEEG^10^, which is supported by the International Neuroinformatics Coordinating Facility (https://www.incf.org/). The structure contains human readable metadata and is widely used by the neuroscience community, enabling automatic processing by a wide range of available tools. The metadata are stored in .tsv and .json files and contain information about anatomical location of implanted electrodes (anatomical structure and coordinates in scanner coordinate space), channel sampling frequencies, channel quality, and behavioral events (e.g. word presentations, recalled words, saccades). The structure of the data follows the BIDS standard version 1.2.1 with the folder levels arranged into a subject-session-iEEG-task-run order. The BIDS runs are individual performances of a particular task by one patient separated by 24–72 hours.

The root dataset folder contains readme and dataset description files with general information about the dataset and participants.tsv describing the metadata of individual patients. The “code” folder contains an ipython notebook with scripts to guide the users and provide easy access to the data. The BIDS subject and session levels do not contain data files, but have structural purpose. The BIDS session level was implemented in case the same patients are reimplanted in the future. The ieeg level contains multiple files and folders directly related to individual recordings. Each run represents one performance of a particular task. The .mefd folders are MEF session folders containing raw data. The file _electrodes.tsv provides information about electrode and contact positions in coordinates and individual anatomical structures. The _channels.tsv files provide information about individual channel type, sampling frequency, filters and data quality. The _events.tsv files provide events that happened during the recording. The events without the trial_type information are the original ttl events as sent by the stimulation laptop. In FR and PAL the events with trial_type information provide detailed description with specific words during encoding and recall along with distractor phase events. In SP the events represent the change in moving dot direction. In AP the events show information about fixation and the following anti/pro-saccade event. The .json files provide general descriptions of the recordings and descriptions of the columns in .tsv files that are not specified in BIDS standard.

The dataset is available under the terms of EBRAINS data usage agreement for human data (Supplement 1) via the EBRAINS Knowledge Graph^41^.

## Technical Validation

The iEEG data were first visually checked for corrupt signals coming from contacts that were either damaged or implanted outside of the brain tissue. The information about the signal quality was stored in the BIDS _channels.tsv files for each subject-task-run. The patient verbal responses were manually marked in the signals using vocal recordings to indicate the start of word vocalization (Fig. 3).

**Fig. 4:**
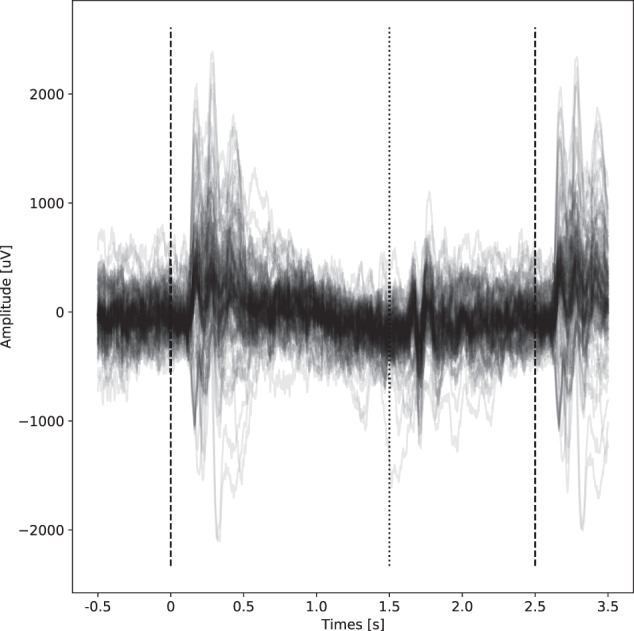
Example of ERPs in FR task. Each line represents a signal averaged across all word presentations from individual channels during the word presentation phase. The dashed lines represent word presentation, the dotted line disappearance of the word from screen.

**Fig. 5:**
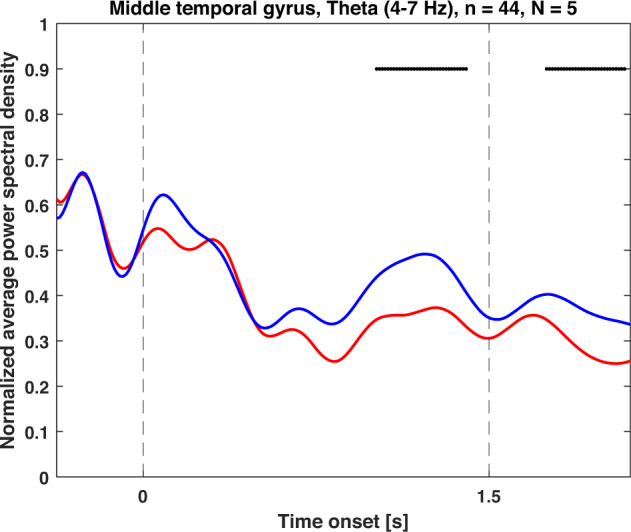
Normalized average power spectral density of theta activity (4–7 Hz) during word presentation in FR. Significant differences (p < 0.05) between recalled (red) and forgotten (blue) words are highlighted by black horizontal lines.

To validate the iEEG signals we first investigated the proper alignment of word presentation with the signals, we averaged the iEEG responses across all trials to confirm clear event-related potentials evoked on a subset of the electrode channels following the word presentation (Fig. 4). To confirm our previous results (Kucewicz *et al.* 2017, 2019)^17,18^ and further confirm the technical validity of iEEG data, we analyzed the changes in the spectral power density in forgotten and recalled words across 44 contacts within 5 patients (Fig. 5). Only patients with electrodes implanted in the middle temporal gyrus were selected. We observed significant differences of this so-called Subsequent Memory Effect in the theta frequency band during presentation of recalled and forgotten words using Kruskal-Wallis, alpha = 0.05.

**Fig. 6:**
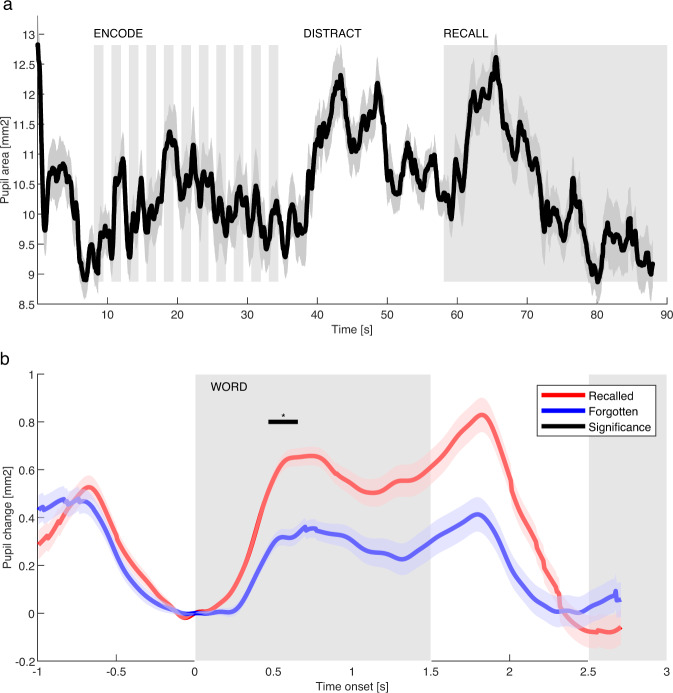
Example of the eye tracking data during FR, sub-004. (**a**) Pupil dilation is modulated by different phases of the free recall verbal memory task. The pupil size was averaged over all trials of one patient in the FR task. Shaded areas mark epochs of word presentation on the screen and their recall with blank screen. (**b**) Recalled and forgotten words show different pupil responses during memory encoding. The pupil responses to word presentation were averaged with respect to subsequently recalled (red) and forgotten (blue) words. Shaded areas mark word presented on the screen.

To validate the synchronization of pupillometry signals and their scientific utility we performed analysis of pupil dilation during a FR task. Pupil dilatation was dynamically modulated during different stages of the task (Fig. 6a). We further evaluated the Subsequent Memory Effect as the difference in pupil dilation during encoding of recalled and forgotten words (Fig. 6b) as reported previously^20^. Significantly larger pupil dilation was observed during presentation of recalled words around 0.5 s after stimulus presentation. Both of these results are congruent with the previous report from a different group of patients (Kucewicz *et al*. 2018)^20^.

## Usage Notes

For easy manipulation with the dataset we recommend usage of BIDS compatible libraries such as pybids^42^ or bids-matlab (https://github.com/bids-standard/bids-matlab) along with the libraries for MEF3 reading mentioned in the data records section. The folder “code” in the dataset root directory contains a jupyter notebook with example code for reading the data in python programming language. The signals are not preprocessed intentionally to provide the data in a raw form and allow the users to perform their own preprocessing. The iEEG signals exhibiting pathological activity are not excluded but can be detected on demand using the provided code (see Code availability). The pupillometry signals contain blinking artifacts and artifacts caused by clinical settings such as movement due to discomfort or pain. The pupillometry signal artifacts can be eliminated by using published methods^43^. The data stored in MEF can be viewed using PySigView^44^, a custom made python viewer, or the SignalPlant program^45^. The iEEG recordings were limited to 128 channels even though the number of implanted contacts was higher. We will provide support to the users if they encounter any problems using the above mentioned methods.

### Limitations

The dataset comprises a relatively small number of recordings. We intend to add recordings over time in new versions of this dataset which will also include parallel recorded electrocorticography (ECoG) data for some cases. Currently the contact coordinate information stored in _electrodes.tsv files are provided in MRI scanner space and is therefore not applicable across patients. Our priority was to provide the anatomical localization data in the raw form to allow conversion of the patient-specific coordinates into a preferred coordinate and anatomical labeling frame, if needed. We will provide support to users if they wish to convert the coordinates to any specific coordinate system. The data set currently does not include the brain images for the sake of protecting patient privacy^46^. This issue will be addressed in the future versions of the dataset. In the new datasets, we are also planning to include micro-contact signal recordings, which present additional challenges for interpolating their coordinates relative to the neighboring macro-contacts.

## Supplementary information

Supplement 1

## Data Availability

To provide an easy way to detect and analyze electrophysiological activity in iEEG signals we make our codes available at GitLab (https://gitlab.com/brainandmindlab/memory_encoding). Currently, the repository contains a python script to automatically process the individual BIDS layers of the dataset (subject, task, run, channel) using the EPYCOM library^47^. The library is focused on iEEG processing and contains a set of algorithms for automated detection of high frequency oscillations^24,48,49^, interictal epileptiform discharges^50^ and for computation of univariate and bivariate feature calculation^51,52^. The repository will be gradually updated with scripts for statistical analyses and result visualizations.
