# Supplementary material for: Intracranial electrophysiological recordings from the human brain during memory tasks with pupillometry
Source: Sci Data. 2022 Jan 13;9:6. doi: 10.1038/s41597-021-01099-z (PMC8758703; doi:10.1038/s41597-021-01099-z)
Supplement: Supplementary file 1 — Supplement 1 [file 41597_2021_1099_MOESM1_ESM.pdf]

EBRAINS Data Use Agreement

version 1.0

Table of Contents

1. Definitions 2
2. Use of Human Data 3

Version

Version	Description	Date
1.0	Initial version of the Data Use Agreement	2020-06-11

This agreement governs the use of pseudonymised human data provided by EBRAINS services to Users. The User agrees to be bound by these terms of use.

1. Definitions

Users: The individual users that access data through EBRAINS.

Data: The data to which the User has access through EBRAINS. Data is used broadly including human data, animal data, or data derived by technical work.

Dataset: Dataset is an identifiable collection of data, either raw or derived, and its associated metadata, including data and metadata derived from monitoring protocols, field observations, collections, laboratory analysis, camera trap images, as well as written, recorded, graphic, audiovisual or other materials in any media. A Dataset may contain software and algorithms.

Data Controller: The natural or legal person, public authority, agency or other body which, alone or jointly with others, determines the purposes and means of the processing of personal data.

Data Providers: The people who provide data to the EBRAINS research platform.

Data Processor: A natural or legal person, public authority, agency or any other body which processes personal data on behalf of the controller.

EBRAINS: The name of the research infrastructure developed by the Human Brain Project.

EBRAINS AISBL: The legal entity (not-for-profit international association) responsible for exploiting the EBRAINS Research Infrastructure.

General Data Protection Regulation (GDPR): Regulation (EU) 2016/679 of the European Parliament and of the Council of 27 April 2016 on the protection of natural persons with regard to the processing of personal data and on the free movement of such data, and repealing Directive 95/46/EC.

Processing: Any operation or set of operations which is performed on personal data including data storage, anonymisation, data transfer, etc.

Personal Data: Data relating to an identified or identifiable natural person.

Project: The project for which the User has accessed these Data.

Pseudonymisation: The processing of personal data in such a manner that the personal data can no longer be attributed to a specific data subject without the use of additional information, provided that such additional information is kept separately and is subject to technical and organisational measures to ensure that the personal data are not attributed to an identified or identifiable natural person.

Publications: Includes, without limitation, articles published in print journals, electronic journals, reviews, books, posters and other written and verbal presentations of research.

Research Participant: An individual whose data form part of these Data.

2. Use of Human Data

In order to use potentially identifiable human Data provided by EBRAINS services, you agree to the following terms:

2.1. You are data controller for any Pseudonymised Data provided by EBRAINS services and will comply with the provisions of the GDPR.
2.2. You will preserve, at all times, the privacy of these Data. In particular, you agree not to attempt to compromise or otherwise infringe the privacy of information on Research Participants.
2.3. You will not attempt to re-identify the Research Participants.
2.4. You will protect the privacy of Research Participants in any research papers or publications that they prepare by taking all reasonable care to limit the possibility of identification.
2.5. You will not attempt to transfer these Data, in whole or part, to anyone beyond your institution.
2.6. You will liaise with the Data Provider with regard to any incidental findings.
2.7. You will destroy/discard the Data held, once it is no longer used for the Project, unless obliged to retain the data for archival purposes in conformity with audit or legal requirements.
2.8. You will distribute a copy of this agreement to Data Processors of the Data. It is the your responsibility to ensure that Data Processors comply with the terms of this agreement.
2.9. You will report any unauthorised access to Data to the EBRAINS Data Protection Officer (data.protection@ebrains.eu) as soon as possible or within 72 hours in compliance with the GDPR.
2.10. Failure to comply to this agreement will result in the termination of access to these Data.
